# Cytological profiling of trypanocidal principles from *Aloe barbadensis* and *Taraxacum officinale*

**DOI:** 10.1016/j.phyplu.2025.100793

**Published:** 2025-05

**Authors:** Pearl Ihuoma Akazue, Neils Ben Quashie, Dorcas Osei-Safo, Sue Vaughan, Harry P. de Koning, Theresa Manful Gwira

**Affiliations:** a West African Centre for Cell Biology of Infectious Pathogens, University of Ghana, Ghana; b Department of Biochemistry, Cell and Molecular Biology, University of Ghana, Ghana; c Department of Biochemistry, Faculty of Life Sciences, University of Benin, Nigeria; d Department of Chemistry, University of Ghana, Ghana; e Department of Biological and Medical Sciences, Oxford Brookes University, UK; f School of Infection and Immunity, University of Glasgow, UK; g Centre for Tropical Clinical Pharmacology and Therapeutics, University of Ghana Medical School, Ghana

**Keywords:** African trypanosomiasis, Antitrypanosomal activity, *Aloe barbadensis*, *Taraxacum officinale*, Mitochondrial oxidative stress, Kinetoplast segregation

## Abstract

The use of herbal medicines to treat ailments is a common practice in several regions in Africa, relying on knowledge systems that have evolved over several generations. These herbal remedies are often based on anecdotal claims, many of which lack scientific validation. This study investigates the mode of action of two bioactive fractions, F1 (IC_50_: 8.5 µg/mL) and F5 (IC_50_: 7.4 µg/mL), derived from a dichloromethane extract of a herbal mixture, consisting of *Aloe barbadensis* and *Taraxacum officinale*, that is commonly used in Ghana to treat parasitic fevers. Both fractions exhibited trypanocidal effects with minimal cytotoxicity to mammalian cells. F5 induced necrotic cell death through mitochondrial oxidative stress, evidenced by a 3.5-fold increase in mitochondrial reactive oxygen species at 2 × IC_50_ (*p*< 0.0001) and significant mitochondrial membrane depolarization (*p*< 0.01). In contrast, F1 primarily disrupted kinetoplast segregation, increasing 2K1 N cells by 3.2-fold at 1 × IC_50_ (*p*< 0.0001) and instigating an accumulation of dyskinetoplastic cells (0KXN). Both fractions induced morphological distortions, nuclear fragmentation, and loss of flagellar integrity. This study provides the first mechanistic insights into the antitrypanosomal activity of bioactive fractions obtained from a mixture of *A. barbadensis* and *T. officinale*. The distinct targeting of mitochondrial ROS production (F5) and kinetoplast replication (F1) highlights their potential as leads for the development of new antitrypanosomal drugs with novel mechanisms of action. These findings reinforce the value of ethnomedicinal plants as sources of novel bioactive compounds.

## Highlights

• F1 and F5 were derived from a mixture of *Aloe barbadensis* and *Taraxacum officinale*.
• F1 and F5 were selectively toxic to trypanosomes via distinct mechanism.
• F5 induced oxidative stress, while F1 caused kinetoplast segregation defects in *T. b. brucei*.
• F1 and F5 disrupted multiple cellular processes, causing necrosis in *T. b. brucei*.

## Graphical abstract

**Figure:**
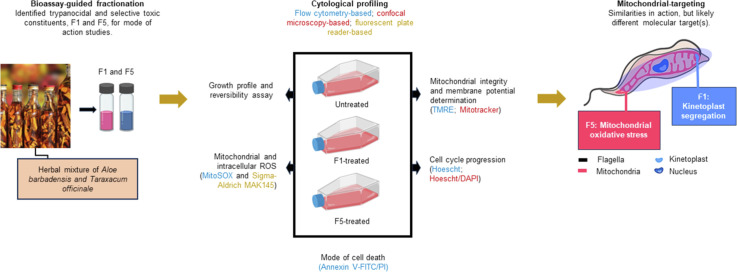

## Introduction

African trypanosomiasis is a vector-borne zoonotic disease that endangers millions of human lives and has devastating effects on sub-Saharan agriculture. The disease is caused by Multiple species of trypanosomes believed to have originated in Africa and were predominantly found there. However, several animal-infective trypanosome species that do not require the tsetse fly vector for transmission are now also widely distributed from South America to the Middle East and parts of Asia (Jones and Davila, 2001; Ungogo and de Koning, 2024). In contrast, Human African Trypanosomiasis (HAT, sleeping sickness) is primarily confined to the tsetse belt (Simarro et al., 2012), with *Trypanosoma brucei gambiense* (gHAT) responsible for the disease in West Africa and *Trypanosoma brucei rhodesiense* (rHAT) causing the disease in the East and parts of Southern Africa (Buscher et al., 2017). While concerted control efforts have led to a significant decline in the incidence of HAT (Akazue et al., 2019; Antillon et al., 2022), Animal African Trypanosomiasis (AAT) remains a persistent challenge leading to substantial livestock losses annually with severe socio-economic consequences (Perry and Grace, 2009; Giordani et al., 2016; Morrison et al., 2016; Isaac et al., 2017).

With the exception of the recently introduced fexinidazole for gHAT, the currently available antitrypanosomal drugs have significant limitations, including unacceptable toxicity profiles and the emergence of drug resistance, which compromises their continued use as first-line therapies (de Koning, 2020). In addition, fexinidazole is only used to treat early and late-stage gHAT, while outdated and highly toxic drugs remain in use for late-stage rHAT (Deeks, 2019; Dickie et al., 2020; de Koning, 2020). There are also rising concerns regarding the long-term efficacy of fexinidazole due to its potential cross-resistance with nifurtimox and other nitroheterocycles (Sokolova et al., 2010; Wyllie et al., 2016; de Koning, 2020). Moreover, unlike HAT, AAT has received comparatively little research attention while the infection continues to spread in livestock populations, exacerbating economic hardships in affected regions (Morrison et al., 2016; Asghari and Rassouli, 2022; Ungogo and de Koning, 2024). Thus, expanding the repertoire of therapeutic leads with improved efficacy and safety profiles for developing new African trypanosomiasis drugs remains a critical research priority.

Medicinal plants have historically served as valuable sources of bioactive compounds for drug discovery, contributing to the development of several modern therapeutics (Newman and Cragg, 2020). However, their use in herbal medicine is often based on anecdotal evidence, and to date only a fraction of ethnomedicinal claims have undergone scientific validation. Yet, bioprospecting approaches have led to the discovery of plant-derived compounds with potencies against diseases beyond their traditional indications. In recent years, several promising plant-derived antitrypanosomal fractions and compounds have been identified based on their ethnopharmacological applications (Hoet et al., 2004; Ibrahim et al., 2014; Ebiloma et al., 2017; Dofuor et al., 2019; Twumasi et al., 2020; Ungogo et al., 2020).

*Taraxacum officinale* (common dandelion) and *Aloe barbadensis* (Aloe vera) are two widely used medicinal plants with a range of pharmacological properties. In Ghana, a combination of these two plants is used to treat parasitic fevers, suggesting a potential anti-parasitic effect that warrants scientific validation. *T. officinale* is a perennial herbaceous plant belonging to the Asteraceae family. It has also been promoted for use in treating constipation, gastrointestinal disorders, and immunodeficient conditions, based on as yet unsubstantiated claims. There are reports of its anti-cancer, anti-diabetic, antifungal, anti-arthritic, antiviral, antibacterial, anti-rheumatoid, hepatoprotective and cardioprotective properties in the scientific literature (Reynolds and Dweck, 1999; Kumar et al., 2019; Sanchez et al., 2020). It contains bioactive compounds such as polyphenols and sesquiterpenes, which may contribute to its observed pharmacological properties (Williams et al., 1996; Bylka et al., 2009; Lee et al., 2011; Martinez et al., 2015; Kamal et al., 2022). Despite these potential benefits, its antiparasitic effects remain largely unexplored.

Similarly, *A. barbadensis*, a succulent herb from the Aloaceae family, is widely regarded as the "healing plant" due to its broad therapeutic applications. It has also been promoted to treat burns, constipation, gastrointestinal disorders, and immune-related conditions based on claims that are yet to be scientifically substantiated. There are also reports of its anti-cancer, anti-diabetic, antifungal, anti-arthritic, antiviral, antibacterial, anti-rheumatoid, hepatoprotective and cardioprotective properties in the scientific literature (Reynolds and Dweck, 1999; Radha and Laxmipriya, 2015; Kumar et al., 2019; Sanchez et al., 2020). *A. barbadensis* contains anthraquinones, polysaccharides, flavonoids, and saponins, which have demonstrated antimicrobial and cytotoxic properties (Kumar et al., 2019; Sanchez et al., 2020). Although its antiplasmodial, and antileishmanial activities have been documented (Abubakar et al., 2006; De Queiroz et al., 2014; Kumar et al., 2017), no prior studies have characterized its active antitrypanosomal activity or their mechanism of action.

Building on the ethnopharmacological relevance of these plants, we have previously reported a dichloromethane extract of a herbal mixture of both plants with significant antitrypanosomal activity. Subsequent bioassay-guided fractionation resulted in the isolation of two semi-purified bioactive fractions, F1 and F5 (Fig. S1), with promising potency against *T. b. brucei* (Twumasi et al., 2020). Building on the previous study, this study aimed to elucidate the mode of action of F1 and F5 on *T. b. brucei* using a cell-based profiling approach. By analyzing the effects of F1 and F5 on key cellular processes and organelles, this study seeks to provide deeper insights into the antitrypanosomal mechanisms of F1 and F5 and reaffirm the potential of herbal medicines as valuable sources of novel anti-infective compounds.

## Materials and methods

### Trypanosome culture

Wild-type bloodstream form *T. b. brucei* GUTat 3.1 strain was cultured in HMI-9 medium (Hirumi and Hirumi, 1994), supplemented with 1 % penicillin-streptomycin (Sigma-Aldrich, Cat No PENNA-100MU and Sigma-Aldrich, Cat No S6501–100 G) and 10 % fetal bovine serum (Gibco, Thermo Fisher Scientific, Waltham, MA, USA; Cat No 10,270,106) at 37 °C and 5 % CO_2_.

### *Aloe barbadensis* and *Taraxacum officinale* fractions

The fractions used in this study, F1 and F5, were obtained via dichloromethane extraction and bioassay-guided fractionation of a dried, pulverised herbal mixture of *A. barbadensis* and *T. officinale* as previously described (Twumasi et al., 2020). To ensure experimental reproducibility, stock solutions of the fractions (20 mg/mL) were prepared in 100 % dimethyl sulfoxide (DMSO; Sigma Aldrich, St. Louis, MO, USA, Cat No D8418) and stored at −20 °C. Working solutions were freshly diluted in autoclaved distilled water at a final concentration of 2 mg/mL before each experiment. All experimental conditions included vehicle controls to account for the potential effects of DMSO, ensuring that the final DMSO concentration did not exceed 0.5 %, a concentration reported to have no adverse effects on the cells (Misuri et al., 2017; Gallardo-Villagran et al., 2022).

### Mammalian cell culture

Murine macrophage cells (RAW 264.7) and human embryonic kidney cells (HEK 293) were maintained at 37 °C and 5 % CO_2_. The cells were cultured in Dulbecco's Modified Eagle's Medium (DMEM; Gibco, Cat No 52,100,039) supplemented with 1 % penicillin-streptomycin and 10 % fetal bovine serum (Gibco, 10,270,106).

### Growth profiling

Growth profiles were used to determine the rate of growth inhibition caused by the fractions on *T. b. brucei* cells, and to gain insight into whether F1 and F5 exhibited trypanostatic or trypanocidal effects. *T. b. brucei* cells in their logarithmic growth phase were diluted to a density of 2 × 10^5^ cells/mL and treated with various concentrations (½ × IC_50_, 1 × IC_50_, 2 × IC_50_, and 4 × IC_50_) of F1 and F5, while untreated cells served as control. The IC_50_ (half-maximal inhibitory concentration) values for F1 and F5 (8.5 µg/mL and 7.4 µg/mL, respectively) were reported in our previous study (Twumasi et al., 2020). These values were used to determine treatment concentrations in subsequent assays to capture sub-lethal, inhibitory, and overtly cytotoxic effects, ensuring a comprehensive assessment of dose-dependent responses. Cells were monitored for up to five days and sub-cultured in fresh HMI-9 medium every 24 h. Daily cell counts were recorded as an index of cell growth and cumulative cell numbers were plotted against time. Doubling time was determined using nonlinear regression analysis (exponential model) in GraphPad Prism version 9 (San Diego, *CA*, USA). Statistical analysis of cell counts at each time point was conducted using two-way ANOVA with Dunnett's multiple comparison test. Data represent the mean of three independent experiments, each with three technical replicates.

### Mitochondrial membrane potential determination

Mitochondrial membrane potential was determined using the tetramethylrhodamine ethyl ester (TMRE) assay, as described by (Eze et al., 2016), with slight modifications. *T. b. brucei* (2 × 10^5^ cells/mL) were treated with various concentrations (½ × IC_50_, 1 × IC_50_, and 2 × IC_50_) of F1 and F5 for 24 h. Cells treated with valinomycin (100 nM) and troglitazone (10 µM) served as negative and positive controls respectively. Troglitazone induces hyperpolarisation of the mitochondrial membrane potential, whereas valinomycin causes depolarisation (Denninger et al., 2007). Treated and untreated trypanosomes were centrifuged at 4500 rpm for 10 min at 4 °C, washed with 1 mL of PBS, and resuspended in 1 mL of PBS containing 200 nM of TMRE (Invitrogen, Cat No T699). The cell suspensions were incubated at 37 °C for 20 min, followed by an additional 30-minute incubation on ice, before acquisition on a Becton Dickinson (BD) FACS Calibur (4-colour) cytometer featuring CellQuest Pro software (BD Biosciences, San Jose, *CA*, USA). TMRE fluorescence was excited at 488 nm and detected using the PE detector. Data represent two independent experiments and were analyzed using FlowJo software version 10.7.1. Statistical analysis (one-way ANOVA with Sidak's multiple comparison test) was conducted using GraphPad Prism version 9.

### Mitochondrial ROS assay

The MitoSOX™ red assay was performed to assess mitochondrial health by measuring mitochondrial reactive oxygen species (ROS) generation. This assay aimed to ascertain whether the observed decrease in mitochondrial membrane potential was due to oxidative stress. The method described by (Dolezelova et al., 2020) was employed with modifications. *T. b. brucei* cells (2 × 10^5^ cells/mL) were treated with F1 or F5 at ½ × IC_50_, 1 × IC_50_ and 2 × IC_50_) of for 1 and 24 h. Valinomycin (500 nM) and troglitazone (10 µM) were used as controls, along with untreated cells. Cells were centrifuged at 4500 rpm for 10 min at 4 °C, resuspended in 1 mL of PBS containing 5 µM of MitoSOX™ red mitochondrial superoxide indicator (Invitrogen, Cat No M36008), and incubated on ice for 30 min. Fluorescence was acquired using a BD FACS LSR Fortessa X-20 flow cytometer with BD FACSDiva software version 8.0.1 (BD Biosciences, San Jose, *CA*, USA). MitoSOX™ red fluorescence was excited using the 488 nm laser and detected using the YG 610/20 filters and 600 LP mirrors for rhodamine A. The assay was conducted in three biological replicates. Data analysis was carried out using FlowJo software 10.7.1. Statistical analysis (repeated measures two-way ANOVA with Sidak's multiple comparison test) was performed using GraphPad Prism version 9.

### Intracellular ROS assay

A one-step red fluorometric intracellular ROS kit (Sigma Aldrich, Cat No MAK145) was used to quantify intracellular ROS, particularly superoxide and hydroxyl radicals, in live cells. *T. b. brucei* cells (2 × 10^4^ cells/well) were treated with F1 or F5 at ½ × IC_50_, 1 × IC_50_ and 2 × IC_50_ for 1 h and 24 h. Valinomycin (500 nM) and troglitazone (10 µM) were included as controls, along with untreated cells. Cells were washed with PBS at 2700 rpm for 10 min, and 100 µL of the master reaction mix prepared as described by the manufacturers was added to each well in a 96-well assay plate. Plates were incubated at 37 °C and 5 % CO_2_ for 1 hour, and fluorescence intensity was measured using a Varioskan™ multimode plate reader (excitation: 520 nm, emission: 605 nm). Background fluorescence was subtracted using blank wells. The assay was performed in three biological replicates. Data and statistical analysis (repeated measures two-way ANOVA with Dunnett's and Sidak's multiple comparison tests) were carried out using GraphPad Prism version 9.

### Microscopy analysis

The effect of F1 and F5 on parasite cell morphology, mitochondrial membrane potential and integrity was investigated using the Mitotracker™ red CMXRos dye (Invitrogen, Thermofischer Scientific, Cat. No M7512), following the protocol described by (Vassella et al., 1997), with slight modifications. *T. b. brucei* cells were treated at ½ × IC_50_, 1 × IC_50_ and 2 × IC_50_ for 24 h, while untreated cells served as controls. Cells were stained with 100 nM of Mitotracker™ red CMXRos dye for 5 min at room temperature. Formaldehyde fixation of bloodstream-form *T. b. brucei* was performed as described by (Dean and Sunter, 2020), with minor modifications. After fixation, cells were counterstained with 0.1 µg/mL of either Hoechst 33,342 (Invitrogen, Cat No H1399) or DAPI (Invitrogen, D1306) before the final PBS wash. Slides were mounted using Vectashield™ antifade mounting medium (Vector Laboratories, Cat No H-1000). Cells were visualized using a Zeiss Axio Observer Z1 Confocal microscope, equipped with an LSM 800 confocal unit, using a 63× oil objective lens with a 1.4 numerical aperture (Plan-Apochromat 63×/1.4 Oil DIC M27) (Carl Zeiss Microscopy GmbH, Jena, Germany). Kinetoplast/nucleus (KN) counts, cell morphology and mitochondrial integrity were analyzed through manual cell counting. Statistical analysis of quantitative cell counts was performed using one-way ANOVA, followed by Sidak's multiple comparison test in GraphPad Prism version 9.

## Results

### F1 and F5 exhibited trypanocidal activity and were non-cytotoxic to mammalian cells

**Fig. 1:**
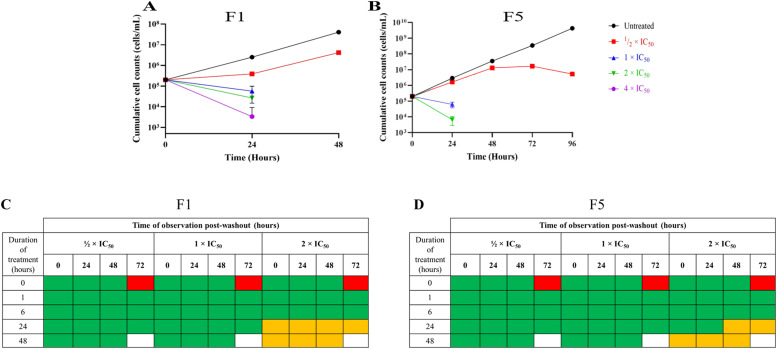
v1. Growth inhibition and washout analysis of *T. b. brucei* treated with F1 and F5. (A) Growth profile of *T. b. brucei* treated with F1 at different concentrations (½ × IC₅₀, 1 × IC₅₀, 2 × IC₅₀, and 4 × IC₅₀) over 48 h. (B) Growth profile of *T. b. brucei* treated with F5 at the same concentrations over 96 h. Cumulative cell counts were determined by multiplying the actual cell counts by the dilution factor. Untreated cells served as controls. Growth inhibition was dose-dependent, with higher concentrations (2 × IC₅₀ and 4 × IC₅₀) leading to significant reductions in cell numbers. (C) Growth reversibility assay for F1-treated *T. b. brucei* cells. Cells were treated with F1 at ½ × IC₅₀, 1 × IC₅₀, and 2 × IC₅₀ for different durations (0, 1, 6, 24, and 48 h), followed by drug removal and monitoring of recovery by microscopy. (D) Growth reversibility assay for F5-treated *T. b. brucei* cells. Cells were treated with F5 at ½ × IC₅₀, 1 × IC₅₀, and 2 × IC₅₀ and observed for recovery under the same conditions. In both (C) and (D), color-coded boxes represent cell viability outcomes: **Green boxes** indicate that cells were observed, signifying recovery and continued growth. **Red boxes** indicate no cells were observed, and cell death was independent of treatment. **Orange boxes** indicate no cells were observed in culture, and cell death was associated with treatment. **Blank boxes** indicate that cells were not observed at that time point. Data represent three biological replicates, each with three technical replicates. Error bars indicate standard deviation (SD).

Untreated *T. b. brucei* cells grew exponentially with a constant doubling time of about 6 h, However, F1 treatment at ½ × IC_50_ led to a significant reduction in the growth rate of the cells after 24 h (*p*= 0.0185) compared to the untreated cells. A further decline in cell numbers was observed at higher concentrations: 1 × IC_50_ (*p*= 0.0143), 2 × IC_50_ (*p*= 0.0151), and 4 × IC_50_ (*p*= 0.0148), indicating the trypanocidal effect of F1 (Fig. 1A). Between 24 and 48 h, cell growth increased (*p*= 0.0077), but only in the ½ × IC_50_ culture and at a lower rate than in the untreated cells (Fig. 1A).

Similarly, F5-treated *T. b. brucei* cells showed a pronounced decline in cell numbers, particularly at 1 × IC_50_ (*p*= 0.0151) and 2 × IC_50_ (*p*= 0.0148) within 24 h (Fig. 1B). At 4 × IC_50,_ no viable cells were detected after 24 h, which appears to indicate a rapid trypanocidal effect. At ½ × IC_50_, growth inhibition occurred more gradually, with a significant reduction in cell numbers observed only after 48 h (*p*= 0.0133). This period of reduced growth was accompanied by an increase in doubling time to 8 h, compared to 6 h in untreated cells. After 48 h, a 24-hour static period was observed followed by a moderate decline in cell numbers at 72 h (Fig. 1B). Washout experiments confirmed that the trypanocidal effects of F1 and F5 were irreversible at 24 h and 48 h when *T. b. brucei* were treated at 2 × IC_50_ (Fig. 1C and D).

To assess whether the observed efficacy of F1 and F5 was due to general cytotoxic effects or selectively toxic against trypanosomes, cytotoxicity assays were performed on murine macrophage (RAW 264.7) and human embryonic kidney (HEK 293) cells (Supplementary Methods). The results showed that F1 and F5 were non-toxic to RAW cells, with CC_50_ (half-maximal cytotoxic concentration) values exceeding 100 µg/mL. Both fractions exhibited high selectivity for trypanosomes with selectivity indices greater than 10 (Table 1). These findings are relevant given that existing antitrypanosomal drugs are known to exhibit significant cytotoxicity in mammalian cells, limiting their therapeutic window.

**Table 1:** Mammalian cytotoxicity determination for F1 and F5.

Treatment	*T. b. brucei* (IC_50_ (µg/mL)	Macrophages (CC_50_ (µg/mL)	Selectivity index
F1	8.50 ± 0.48^#^	>100	>11.76
F5	7.37 ± 0.83^#^	>100	>13.57
PAO	<0.20	0.98 ± 0.14	>4.90
DA	0.13 ± 0.02^#^	36.54 ± 1.92	281.08

Values are Mean ±SD (where applicable). Means are averages of three biological replicates, each with three technical replicates. Diminazene aceturate (DA) is a standard antitrypanosomal drug. Phenylarsine oxide (PAO) is a standard cytotoxic drug which served as the positive control. The selectivity index is CC_50_ (from mammalian cytotoxicity screening) divided by IC_50_ (from antitrypanosomal screening). ^#^IC_50_ values for these fractions had earlier been reported (Twumasi et al., 2020).

### F1 and F5 induced necrotic cell death

**Fig. 2:**
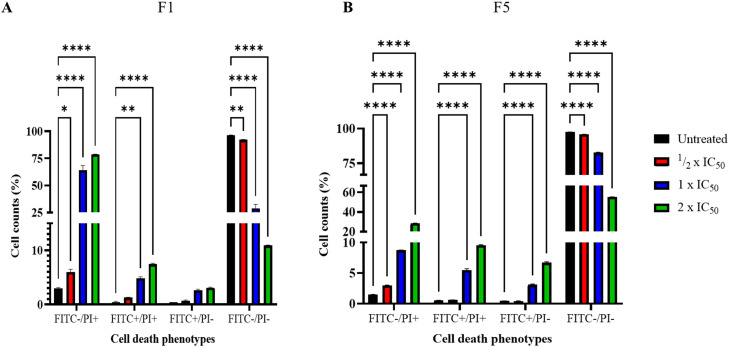
F1 and F5 induce necrotic cell death in *T. b. brucei*. Flow cytometry analysis of Annexin V-FITC/Propidium iodide (PI)-stained trypanosomes treated with F1 (A) and F5 (B) at ½ × IC_50_, 1 × IC_50_, and 2 × IC_50_ for 24 h. Untreated cells served as controls. Cell death phenotypes were classified as follows: live cells (FITC-/PI-, indicating intact membranes and viability), early apoptotic cells (FITC+/PI-, indicating phosphatidylserine externalization without membrane compromise), late apoptotic/necrotic cells (FITC+/PI+, indicating either late-stage apoptosis or secondary necrosis), and necrotic cells (FITC-/PI+, indicating loss of membrane integrity without Annexin V binding, characteristic of primary necrosis). Both F1 and F5 significantly increased the proportion of necrotic (FITC-/PI+) and late apoptotic/necrotic (FITC+/PI+) cells in a dose-dependent manner, with F5 exhibiting stronger effects at lower concentrations. Data represent the mean ± SEM from three independent experiments. Statistical significance was determined using one-way ANOVA with Sidak's multiple comparison test. *p≤ 0.05, **p≤ 0.01, ****p≤ 0.0001. See Supplementary Fig. S3 and S4 for representative pseudocolour two-parameter density plots.

To evaluate the mode of cell death, flow cytometry analysis was performed to quantify the proportion of early apoptotic-like, late apoptotic-like and necrotic *T. b. brucei* cells treated with various concentrations of F1 (Fig. 2A) and F5 (Fig. 2B). F1 treatment did not significantly alter early apoptotic *T. b. brucei* cell proportions at any tested concentration (Fig. 2A). However, a significant increase in late apoptotic-like/necrotic cells was observed at 1 × IC_50_ (*p* = 0.0026) and 2 × IC_50_ (*p* < 0.0001), but not at ½ × IC_50_. Additionally, F1 treatment resulted in a significant increase in necrotic cells at all tested concentrations: ½ × IC_50_ (*p* = 0.046), 1 × IC_50_ (*p* < 0.0001) and 2 × IC_50_ (*p* < 0.0001), which might indicate that necrosis is a dominant mode of parasite death for F1. F5 treatment significantly increased necrotic cells at ½ × IC_50_ (*p*< 0.0001), while early apoptotic-like cells and necrotic/late apoptotic-like cells remain unchanged (Fig. 2B). However, at 1 × IC_50_ and 2 × IC_50_, significant increases in necrotic (*p*< 0.0001), late apoptotic-like/necrotic (*p*< 0.0001), and early apoptotic-like cells (*p*< 0.0001) were observed. These findings suggest that both F1 and F5 induce necrotic cell death in *T. b. brucei,* rather than classical apoptosis, which is consistent with necrotic effects observed in melarsoprol-treated trypanosomes (Wyllie et al., 2016)*.*

### F1 and F5 significantly decreased mitochondrial membrane potential

**Fig. 3:**
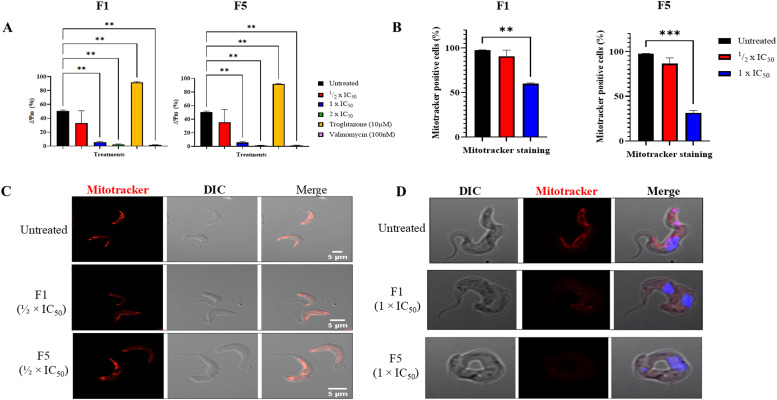
F1 and F5 induce mitochondrial membrane depolarization in *T. b. brucei*. (A) Quantification of mitochondrial membrane potential (ΔΨm) in *T. b. brucei* treated with F1 and F5 at ½ × IC_50_, 1 × IC_50_, and 2 × IC_50_ for 24 h. Cells were stained with tetramethylrhodamine ethyl ester (TMRE), and mitochondrial depolarization was analyzed by flow cytometry. Valinomycin (100 nM) served as a negative control (mitochondrial depolarization), while Troglitazone (10 μM) was used as a positive control (mitochondrial hyperpolarization). Data represent the means of two independent experiments. Statistical significance: *p*≤ 0.01 (**), *p*≤ 0.001 (***), *p*≤ 0.0001 (****). Error bars represent the standard deviation (SD). (B) Proportion of Mitotracker-positive cells following treatment with F1 and F5. Cells were classified as Mitotracker-positive if they exhibited a continuous mitochondrial signal (linear, looped, branched, or complex) extending through the entire cell body, characteristic of normal mitochondrial morphology. At least 200 cells per treatment were counted across three independent experiments. (C, D) Representative confocal micrographs of Mitotracker-stained cells showing mitochondrial morphology in untreated and treated trypanosomes. Mitotracker Red (red) marks the mitochondrion, while Hoechst (blue) stains the nucleus and kinetoplast. (C) At ½ × IC_50_, some mitochondrial signal is retained. (D) At 1 × IC_50_, Mitotracker staining is severely reduced, confirming mitochondrial depolarization. Untreated cells served as controls. All treatments were for 24 h.

To investigate the effects of F1 and F5 on the mitochondrial membrane potential, flow cytometric analysis of TMRE-stained *T. b. brucei* cells was conducted (Fig. 3). Consistent with previous studies (Denninger et al., 2007; Ibrahim et al., 2011), troglitazone and valinomycin induced mitochondrial hyperpolarisation and depolarization, respectively (Fig. 3A). Similarly, both F1 and F5 caused mitochondrial membrane depolarisation at 1 × IC_50_ and 2 × IC_50_. These observations were further validated by confocal microscopy analysis of Mitotracker-stained cells, which revealed reduced mitochondrial fluorescence intensity in F1- and F5-treated cells (Fig. 3B–D). These mitochondrial alterations are similar to those induced by nifurtimox and fexinidazole, both of which exert their trypanocidal effects through mitochondrial dysfunction (Sokolova et al., 2010). However, F1 and F5 appear to induce these effects with distinct kinetics as evidenced by differences in the timing and progression of mitochondrial depolarization, suggesting potential differences in their precise mechanism.

### F1 and F5 significantly increased mitochondrial ROS levels but did not affect overall intracellular ROS levels

**Fig. 4:**
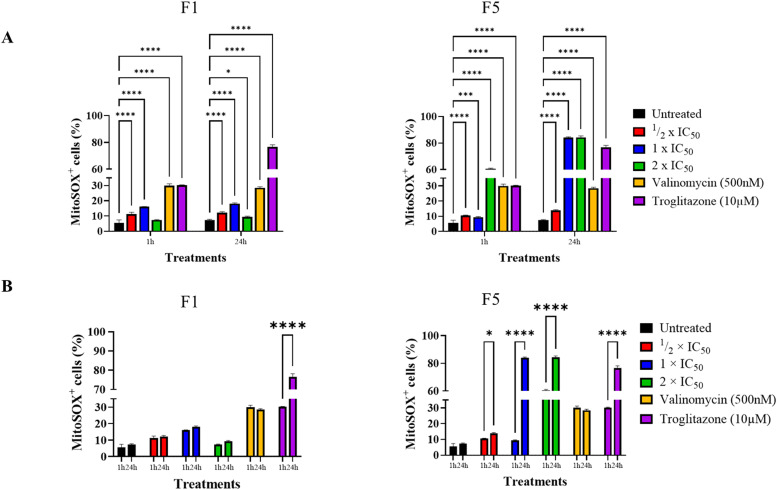
F1 and F5 increase mitochondrial ROS production in *T. b. brucei*. (A) Quantification of mitochondrial superoxide levels in *T. b. brucei* treated with F1 and F5 for 1 hour and 24 h at ½ × IC_50_, 1 × IC_50_, and 2 × IC_50_, as detected using MitoSOX Red staining and flow cytometry. Untreated trypanosomes and trypanosomes treated with valinomycin (500 nM) and Troglitazone (10 μM) were controls. Pairwise comparisons were made with the untreated control at each time point. (B) Time-dependent effects of F1 and F5 on mitochondrial ROS accumulation. The proportion of MitoSOX+ cells was compared between 1 h and 24 h for each treatment, including the untreated control. F5 induced a significant, dose-dependent increase in mitochondrial ROS, while F1 exhibited a more variable response. Statistical significance: *p*≤ 0.05 (*), *p*≤ 0.001 (***), *p*≤ 0.0001 (****). Error bars indicate standard deviation (SD). Data represent three independent biological replicates.

The MitoSOX red assay was used to examine oxidative stress induction in *T. b. brucei* cells treated with various concentrations of F1 and F5. Mitochondrial ROS levels were significantly elevated after 1 h and 24 h of treatment in all treated cells (*p* < 0.0001), except for cells treated with F1 at 2 × IC_50_ after 1 h (Fig. 4A). However, at 2 × IC_50_ after 24 h, F1 caused a significant increase in mitochondrial ROS levels (*p*= 0.0346) compared to untreated cells. The increase in mitochondrial ROS levels for F5 appeared to be more clearly dose-dependent, with significantly elevated ROS levels at all concentrations (½ × IC_50_: *p*= 0.0043; 1 × IC_50_ and 2 × IC_50_: *p*< 0.0001 for both) (Fig. 4A). Mitochondrial ROS levels in valinomycin-treated cells, highly significantly elevated at 1 h (*p*< 0.0001), remained unchanged between 1 h and 24 h, in keeping with valinomycin being a very fast acting drug, strongly depolarizing the mitochondrial membrane in 30 min (Alkhaldi et al., 2016). Conversely, troglitazone treatment caused a significant increase in mitochondrial ROS levels at 1 h and a further strong increase at the 24 h point (*p*< 0.0001); the effects of troglitazone on the mitochondrial membrane potential peak around the 8 h mark (Alkhaldi et al., 2016) (Fig. 4A and B).

**Fig. 5:**
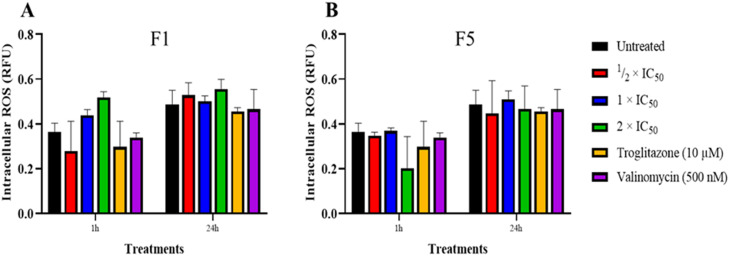
Effect of F1 and F5 on intracellular reactive oxygen species (ROS) levels in *T. b. brucei*. (A) Intracellular ROS levels in *T. b. brucei* following treatment with F1 at ½ × IC_50_, 1 × IC_50_, and 2 × IC_50_ for 1 h and 24 h, measured using a fluorometric intracellular ROS detection assay. (B) Intracellular ROS levels in *T. b. brucei* following treatment with F5 under the same conditions. Trypanosomes treated with troglitazone (10 μM), valinomycin (500 nM) served as controls. Untreated cells served as a baseline reference. No significant increase in intracellular ROS was observed in F1- or F5-treated cells compared to untreated controls, suggesting that mitochondrial ROS accumulation did not translate into widespread oxidative stress in the cytosol. Mean intracellular ROS levels are expressed in relative fluorescence units (RFU). A pairwise comparison was performed against untreated controls at each time point. Error bars represent standard deviation (SD). Data represent three independent biological replicates.

Despite the significant increase in mitochondrial ROS levels, the overall intracellular ROS levels remained unaltered in all F1- and F5-treated cells after 1 h and 24 h (Fig. 5A and B). These findings suggest that F1 and F5 induce mitochondrial oxidative stress in *T. b. brucei.* This mechanism is similar to that of nifurtimox and melarsoprol, both of which have been reported to increase mitochondrial ROS levels (Wilkinson et al., 2008). While nifurtimox undergoes nitroreduction by trypanosome-specific nitroreductases (Hall et al., 2011) to generate cytotoxic free radicals, melarsoprol disrupts trypanothione metabolism leading to oxidative stress. The precise metabolic pathways through which F1 and F5 elevate mitochondrial ROS levels will need to be established.

### F1 and F5 altered cell cycle progression and kinetoplast segregation

**Fig. 6:**
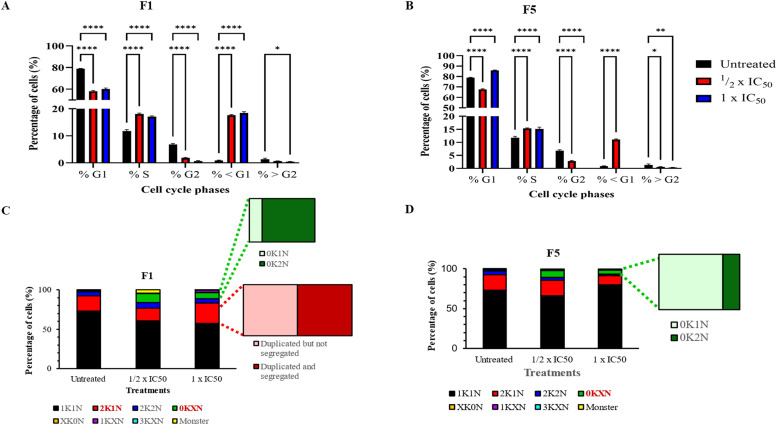
Effects of F1 and F5 on cell cycle progression and kinetoplast/nucleus (K/N) configurations in *T. b. brucei*. (A-B) Bar charts showing flow cytometry analysis of cell cycle distribution in F1-treated (A) and F5-treated cells (B) at ½ × IC₅₀ and 1 × IC₅₀ for 24 h. The percentage of cells in the G1, S, G2, <G1, and >G2 phases is shown for three biological replicates. Untreated cells served as controls. Significant differences compared to untreated controls are indicated (*p≤ 0.05, **p≤ 0.01, ****p≤ 0.0001). At least 500 cells were analyzed per treatment. (C and D) Confocal microscopy analysis of Hoechst-stained cells showing kinetoplast/nucleus (K/N) configurations in F1-treated (C) and F5-treated (D) cells. Stacked bar graphs represent the proportion of cells exhibiting different K/N phenotypes: 1K1N, 2K1N, 2K2N, 0KXN, XK0N, 1KXN, and 3KXN populations. 0KXN cells include 0K1N, 0K2N, and 0K3N phenotypes (no kinetoplast, one or more nuclei). XK0N cells include 1K0N and 2K0N phenotypes (one or more kinetoplast, no nuclei). 1KXN cells include 1K2N and 1K3N phenotypes (single kinetoplast, multiple nuclei). 3KXN cells include 3K1N and 3K2N phenotypes (three kinetoplasts, one or two nuclei). Monster cells were abnormally large multinucleated and/or multikinetoplast cells likely resulting from defective cytokinesis or disrupted segregation of the kinetoplast/nuclei. Insets highlight 2K1N cells (including those with duplicated but not segregated kinetoplasts) and 0KXN cells. All treatments were conducted for 24 h, and at least 500 cells were counted per replicate.

In this study, the effects of F1 and F5 on cell cycle progression were assessed using flow cytometric sorting of Hoechst-stained cells to quantify the DNA content of the cells (Fig. 6A and B). In F1-treated cells at ½ × IC_50_ and 1 × IC_50,_ a significant reduction in the G1 (*p*< 0.0001) and G2 (*p*< 0.0001) phase populations was observed, alongside a corresponding increase in S-phase and < G1 phase cells (Fig. 6A). Similarly, F5 treatment at ½ × IC_50_ led to a significant decrease in G1 and G2 phase cells (*p*< 0.0001), with a corresponding increase in S-phase (*p*< 0.0001) and < G1 phase sells (*p*< 0.0001) (Fig. 6B). However, at 1 × IC_50_, the G1 phase and S-phase population increased (*p*< 0.0001), while G2 (*p*< 0.0001) and < G1 phase cells (*p*= 0.0004) decreased.

Cell cycle analysis by confocal microscopy confirmed these findings (Fig. 6C and D). A significant decrease in the 1K1N population was observed in F1-treated cells at ½ × IC_50_ (*p*= 0.0005) and 1 × IC_50_ (*p*< 0.0001), and in F5-treated cells at ½ × IC_50_ (*p*= 0.0035), compared to untreated controls (Fig. 6C). At 1 × IC_50_, F5 treatment led to an increase in 1K1N cells (*p*= 0.0068) and a decrease in 2K1N cells (*p*= 0.0020) (Fig. 6D). The 0KXN population (consisting of 0K1N, 0K2N and 0K3N cells) increased significantly in cells treated with F1 and F5 at ½ × IC_50._ However, this increase in the 0KXN population was not observed at 1 × IC_50_ (Fig. 6D). Most of the untreated cells and cells treated with F5 at ½ × IC_50_ in the 0KXN population had the 0K1N phenotype, while F1-treated cells had the 0K2N population (Fig. 6C and D). At 1 × IC_50_, F1-treated cells marked as 0KXN were mostly 0K1N cells, while no 0K2N cells were observed. Interestingly, F1-treated cells exhibited an accumulation of 2K1N cells with duplicated but not segregated kinetoplasts (Fig. 6C), suggesting a kinetoplast segregation defect might have occurred despite nuclear division occurring.

**Fig. 7:**
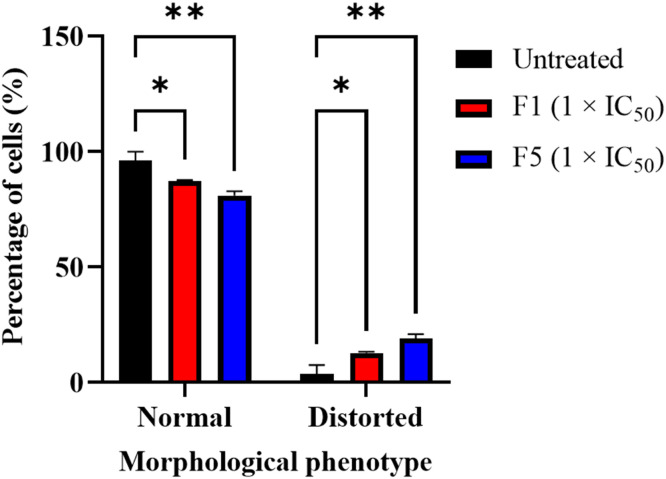
Effect of F1 and F5 on *T. b. brucei* cell morphology. Bar chart showing the proportion of *T. b. brucei* cells exhibiting normal and distorted morphological phenotypes following treatment with F1 (1 × IC₅₀) and F5 (1 × IC₅₀) for 24 h, as determined by differential interference contrast (DIC) microscopy. Morphological distortion was assessed based on deviations in cell shape, size, and flagellar morphology. At least 200 cells were counted per experiment, with data representing the mean of two independent experiments. Untreated cells served as controls. Both F1 and F5 significantly increased the proportion of distorted cells, with a corresponding decrease in normal cells compared to untreated controls. Statistical significance was determined using one-way ANOVA with Sidak's multiple comparison test. Statistical significance: *p*≤ 0.05 (*), *p*≤ 0.01 (**). Error bars indicate standard deviation (SD).

Additionally, both F1- and F5-treated cells displayed morphological abnormalities at higher concentrations (Fig. 7). Pentamidine is known to selectively accumulate in the mitochondrion of *T. brucei* disrupting kinetoplast DNA replication and ultimately leading to kinetoplast loss – dyskinetoplastic cells. Here, F1 and F5 might act through a different mechanism that in most cases affects kinetoplast segregation and/or associated processes but also results in a minor population of dyskinetoplastic cells.

## Discussion

Plants have long served as valuable sources of new therapeutic agents, including treatments for parasitic diseases. Several natural products with antitrypanosomal activity have been identified from various plant sources (Suzuki et al., 2015; Ebiloma et al., 2017; Ohashi et al., 2018; Dofuor et al., 2019) and propolis (Ebiloma et al., 2020), a material bees make from plant resins. In a previous study, we identified two bioactive fractions, F1 and F5, from a herbal mixture containing *A. barbadensis* and *T. officinale,* which exhibited promising antitrypanosomal activity using a bioassay-guided isolation approach (Twumasi et al., 2020). In the current study, we further investigated the potential mode of antitrypanosomal action of these fractions using cytological profiling. This approach provides rapid insights into the cellular processes and organelles targeted by drug treatments, making it a valuable tool for understanding antiparasitic mechanisms (Tomas and Castro, 2013).

Both F1 and F5 exhibited irreversible trypanocidal activity, a desirable characteristic in drug development, as it reduces the likelihood of persister populations that could lead to drug resistance (Fairlamb et al., 2016). The rapid inhibition of parasite growth observed in F5-treated cells appears to be linked to its mitochondrial toxicity, as evidenced by a significant increase in mitochondrial ROS levels. Mitochondrial ROS can disrupt essential metabolic processes by permeating the inner mitochondrial membrane and affecting cytosolic organelles (Tomas and Castro, 2013). However, F5-induced oxidative stress remained largely confined to the mitochondria, as cytosolic ROS levels remain unchanged. This finding suggests either the mitochondrial membrane remained intact or cytosolic antioxidant defence mechanisms mitigated oxidative stress from a possible breach in the mitochondrial membrane. This observation is consistent with previous findings by (Maharjan et al., 2014), which suggest that mitochondrial oxidative stress precedes cytosolic oxidative stress. The results of the TMRE flow cytometry for ΔΨm, and the mitotracker staining, does suggest that a significant proportion of cells has lost mitochondrial function after treatment with either fraction.

Given that nifurtimox and melarsoprol—two frontline antitrypanosomal drugs—promote superoxide production, and fexinidazole, the recently introduced oral nitro drug for gHAT treatment, is believed to function similarly (Fairlamb et al., 1989; Prathalingham et al., 2007), the mitochondrial perturbations observed in F5-treated cells may represent a key trypanocidal mechanism warranting further investigation. Unlike nifurtimox and fexinidazole, however, F5-induced ROS accumulation appears to be more selective for the mitochondrion – a potential mechanistic distinction which might indicate a more targeted mechanism of mitochondrial perturbation that requires further validation. Cross-resistance with the synthetic nitro-heterocycle drugs is unlikely to occur as these depend on activation by strong nitroreductases (Hall et al., 2011) and nitro compounds do not occur in natural plant extracts.

So far, the mitochondrial toxicity (mito-toxicity) of F5 appears to be the most logical explanation for its rapid trypanocidal effects. This phenomenon is reminiscent of oxidative stress-mediated rapid growth inhibition observed in *T. b. gambiense* (Yokoyama et al., 2021). Moreover, mammalian cytotoxicity assays demonstrated that both F1 and F5 were selectively toxic to *T. b. brucei* while exhibiting minimal toxicity towards mammalian cells, at least *in vitro*. The selective toxicity of ROS-generating antitrypanosomal treatments is biologically plausible, given that trypanosomes possess distinct ROS-scavenging machinery and mitochondrial metabolism compared to their mammalian hosts. Unlike mammalian cells, the long-slender bloodstream form *T. brucei* lacks most of the canonical Krebs cycle and electron transport chain, relying instead on a Trypanosome Alternative Oxidative system for energy generation (Ebiloma et al., 2018, 2019). Consequently, perturbations in mitochondrial redox homeostasis are more likely to be catastrophic for *T. b. brucei* cells but less detrimental to their host cells, making mitochondrial disruption a valuable therapeutic target. Furthermore, the observed mitochondrial membrane depolarization in F5-treated cells, along with its likely interference with kinetoplast duplication and/or segregation, suggests that mitochondrion-based oxidative stress might be a key mediator of its trypanocidal activity or might be the result of an action affecting the kinetoplast or be the cause of the kinetoplast effects observed.

Both F1 and F5 induced mitochondrial membrane depolarization, a phenomenon associated with mitochondrial dysfunction. This loss of mitochondrial membrane potential could be a direct result of oxidative stress or a secondary consequence of ATP depletion leading to necrotic cell death. Previous studies have reported ROS-mediated necrotic cell death in trypanosomes (Ridgley et al., 1999), supporting our hypothesis that mitochondrial dysfunction is likely a key driver of F5-induced cell death. Alternatively, the observed mitochondrial membrane depolarisation could stem from a depletion of energy (ATP) levels, which would require further investigation through ATP depletion assays to establish whether F5 primarily disrupts mitochondrial energy production, or if ROS accumulation itself drives cell death.

In addition to the critical roles of the mitochondrion in energy metabolism and cell signalling, it is also essential for cell cycle progression in trypanosomes, and the replication of kinetoplast DNA is initiated before nuclear S-phase, and kinetoplast segregation must be completed before the start of mitosis – making the trypanosome nuclear cell cycle dependent on correct completion of the kinetoplast replication cycle (McKean, 2003). Our data suggest that F1 may interfere with kinetoplast segregation, as evidenced by an accumulation of cells with incompletely segregated kinetoplasts, which could lead to daughter cells with two or no kinetoplasts, respectively, after uneven cell division. It is also possible that F1 not only prevented kinetoplast segregation but also cause uneven cell division. Kinetoplast-targeting effects have previously been described for pentamidine, a standard HAT drug which progressively induces kinetoplast loss in *T. b. brucei* (Thomas et al., 2018). Pentamidine and other diamidine trypanocides selectively accumulate in the mitochondrion and target kinetoplast DNA ultimately leading to dyskinetoplastic cells (Stewart et al., 2005; Robertson et al., 2021). In contrast, F1 appears to interfere with kinetoplast segregation without causing complete kinetoplast loss. Similar kinetoplast-targeting effects have been reported for the veterinary phenanthridine trypanocides, isometamidium and ethidium, both of which interfere with kinetoplast DNA integrity (Roy Chowdhury et al., 2010; Eze et al., 2016). The kinetoplast, a highly specialized mitochondrial sub-organelle, is thus a known target for several antitrypanosomal compounds in clinical or veterinary use. For instance, ethidium bromide disrupts kinetoplast replication by binding directly to DNA minicircles or interference with kinetoplast-associated proteins (Roy Chowdhury et al., 2010). However, the effects of F1 on kinetoplast integrity may well arise from an alternative yet undetermined pathway. A significant increase in 1K1N and 0K1N cells at higher concentrations of F5 suggests inhibition of kinetoplast duplication, although nuclear division and cytokinesis remained arrested. To gain deeper insights into the effects of F1 and F5, follow-up experiments, including live-cell imaging to track kinetoplast replication and segregation in real-time, will be necessary.

Notably, the observed effects of F1 and F5 on *T. b. brucei* cells were dose-dependent, an important consideration for future pharmacokinetic and toxicity studies. Higher concentrations led to greater accumulation of dyskinetoplastic cells and more pronounced reduction in mitochondrial membrane potential, suggesting potential therapeutic thresholds that must be carefully optimized to balance efficacy and selectivity. Establishing the optimal therapeutic window for F1 and F5 will be critical for minimizing off-target toxicity while maximizing trypanocidal potency.

The observed morphological changes, including flagellar defects, could be a direct or indirect consequence of mitochondrial perturbations or defects in kinetoplast segregation. Although bloodstream form *T. b. brucei* can survive without a kinetoplast, dyskinetoplastic cells must acquire compensatory mutations in the nuclear DNA-encoded γ-subunit of the F_o_F_1_-ATPase to sustain ATP production and survive (Schnaufer et al., 2002; Eze et al., 2016). When these adaptations fail to occur, cell death becomes inevitable, which might explain the effects of both F1 and F5 on the treated cells. Overall, these findings indicate that F1 and F5 may target different yet interconnected mitochondrial processes.

A key question that remains unanswered is whether F1 and F5 share cross-resistance mechanisms with existing kinetoplast-targeting drugs, based on the apparent kinetoplast-destabilizing effects observed in F1- and F5-treated cells. Given that pentamidine, diminazene aceturate, and isometamidium target DNA-binding and mitochondrial processes, assessing cross-resistance in trypanosome strains resistant to these compounds would be crucial for determining their long-term therapeutic viability. However, it should be noted that resistance to the diamidine drugs diminazene and pentamidine, at least, is not linked to mitochondrial or kinetoplast changes in *T. brucei*, but to the functional loss of specific transporters TbAT1 and TbAQP2 for these drugs (Munday et al., 2015; Alghamdi et al., 2020). Since these transporters have a narrowly defined substrate profile (Carter et al., 1999; Alghamdi et al., 2020) the chance of cross-resistance is low and in many studies with phytochemicals form plants and propolis we have not observed such cross-resistance with natural compounds.

Additionally, because F1 and F5 are complex mixtures, the observed multi-target effects may arise from individual or synergistic actions of multiple constituents. F5 contains about six detectable compounds, while F1 has yielded seven isolated constituents, all of which were inactive against *T. b. brucei* (Twumasi et al., 2020). However, an oil derived from F1 (F1/HML) exhibited over 1.3-fold greater activity than diminazene aceturate, a standard drug for treating animal trypanosomiasis. This suggests that the active principles responsible for the observed antitrypanosomal effects are either present in minor amounts or require synergistic interactions to exert their full potency. Ongoing work is focused on re-extracting and isolating the active principles from both fractions, particularly from the oily residues, which have previously shown promising activity (Twumasi et al., 2020).

Our findings suggest that F1 and F5, derived from a herbal mixture of *T. officinale* and *A. barbadensis,* exert distinct trypanocidal effects: F5 primarily induces mitochondrial oxidative stress, whereas F1 disrupts kinetoplast segregation. To our knowledge, we provide the first report on the antitrypanosomal activity of these plant species (Twumasi et al., 2020) and characterize their bioactive fractions, providing insights into their mode of action. These results highlight the potential of these fractions as novel therapeutic leads for developing new antitrypanosomal treatments, warranting further mechanistic, pharmacokinetic and *in vivo* validation.

While this study provides key insights into the trypanocidal mechanisms of F1 and F5, certain limitations should be acknowledged. Firstly, the observed activity was assessed using semi-purified fractions rather than isolated compounds, making it necessary to further characterize the specific bioactive constituents responsible for the observed effects. Additionally, as the findings are based on *in vitro* assays, their therapeutic relevance requires further validation using *in vivo* models of African trypanosomiasis. Thirdly, the selectivity of F1 and F5 against a broader panel of mammalian cell lines would be necessary to ascertain their safety profile. Future research will focus on isolating and structurally characterizing the active compounds within F1 and F5 to determine their precise molecular targets, conducting preclinical *in vivo* studies to assess their efficacy and safety, and performing cross-resistance profiling in pentamidine- and diminazene-resistant *T. brucei* strains to assess their potential as therapeutic candidates. Investigating potential resistance mechanisms in *T. b. brucei* will be crucial for determining the long-term utility of these fractions as drug leads. These next steps will provide a more comprehensive evaluation of the potential of *A. barbadensis* and *T. officinale*-derived compounds in antitrypanosomal drug development.

## CRediT authorship contribution statement

**Pearl Ihuoma Akazue:** Writing – review & editing, Writing – original draft, Visualization, Validation, Methodology, Investigation, Funding acquisition, Formal analysis, Conceptualization. **Neils Ben Quashie:** Writing – review & editing, Writing – original draft, Visualization, Validation, Supervision, Formal analysis. **Dorcas Osei-Safo:** Writing – review & editing, Writing – original draft, Resources, Formal analysis, Conceptualization. **Sue Vaughan:** Writing – review & editing, Writing – original draft, Supervision, Resources, Funding acquisition, Formal analysis. **Harry P. de Koning:** Writing – review & editing, Writing – original draft, Visualization, Supervision, Formal analysis. **Theresa Manful Gwira:** Writing – review & editing, Writing – original draft, Visualization, Validation, Supervision, Resources, Project administration, Funding acquisition, Formal analysis, Conceptualization.

## Declaration of competing interest

The authors declare that they have no known competing financial interests or personal relationships that could have appeared to influence the work reported in this paper.

## Appendix Supplementary materials

Image, application 1
