# Supplementary material for: Cytological profiling of trypanocidal principles from Aloe barbadensis and Taraxacum officinale
Source: Phytomed Plus. 2025 May;5(2):None. doi: 10.1016/j.phyplu.2025.100793 (PMC12064442; doi:10.1016/j.phyplu.2025.100793)
Supplement: Supplementary file 1 [file mmc1.docx]

**Methods**

**Growth reversibility assay**

Qualitative washout experiments were used to assess the reversibility of antitrypanosomal action of the fractions after continuous exposure of the fractions to *T. b. brucei* cells at different durations. This assay was to confirm whether the mode of growth inhibition following treatment with the fractions was reversible or irreversible. *T. b. brucei* cells (1 × 10^6^ cells) exposed to various concentrations (½ × IC_50,_ 1× IC_50_ and 2 × IC_50_) of the fractions were harvested at definite time points (1, 6, 24, 48 and 72 hours) by centrifuging at 2,700 rpm (800 g) for 10 minutes. Untreated cells were also harvested and served as the control. The harvested cells were washed twice with Voorheis’ modified PBS (vPBS), resuspended in fresh media in a 24-well plate and observed by microscopy. The cells were monitored every 24 h for up to 72 h.

**Cytotoxicity testing**

A resazurin-based assay was used for the mammalian cytotoxicity screening of the fractions. The mammalian cells were seeded at 1 × 10^4^ cells/well, except for the blank control wells which had no cells, and left for 24 hours. Serial dilutions of the fractions in DMEM were made in a separate plate and aliquoted into the assay plate containing the seeded cells, except for the wells containing the untreated control cells. The final DMSO concentration in each of the wells did not exceed 0.5%. After incubating for 24 hours, 20 µL of the Alamar blue dye solution (500 µM of resazurin sodium salt in PBS) was added to each well and incubated for an additional 24 hours. Cells treated with phenyl arsine oxide (PAO; Sigma), a standard cytotoxic drug, served as the positive control as described (Zheoat et al., 2021). Fluorescence readings were taken using a Varioskan™ multimode plate reader at excitation wavelength 530 nm and emission wavelength 590 nm. Plate readouts were analysed using non-linear regression analysis for growth inhibition on GraphPad Prism version 9, and CC_50_ values were determined. The assay was performed in three independent biological replicates, with each biological replicate containing three technical replicates.

**Mode of cell death assay**

There is an ongoing discussion concerning the occurrence of true apoptotic cell death in trypanosomes due to the absence of caspases and other receptors for apoptotic cell death signalling pathways (Kaczanowski et al., 2011). However, though trypanosomes lack caspases and apoptotic signalling receptors, apoptotic-like cell death can be observed as evidenced by phosphatidylserine externalisation, which could give insights into cell death mechanisms.

In the presence of Ca^2+^, annexin V conjugated with FITC typically binds to phosphatidylserine. Phosphatidylserine is usually found in the inner plasma membrane of healthy cells. In the early stages of apoptosis, phosphatidylserine becomes externalised, making it easily accessible for annexin V-FITC to bind to it; propidium iodide (PI) is a fluorescent dication that is excluded from intact trypanosomes but enters when the plasma membrane becomes compromised, and then increases fluorescence from interaction with nucleic acids (Gould et al., 2008; Ibrahim et al., 2011). As a result, the FITC+/PI- population are early apoptotic-like cells. In the late stages of apoptosis, the plasma membrane becomes compromised, allowing PI to bind to nucleic acids resulting in FITC/PI-double positive cells (FITC+/PI+). On the other hand, during necrotic cell death, the plasma membrane ruptures, allowing for PI staining; hence, the PI+ populations (FITC-/PI+ and FITC+/PI+) represents necrotic cells. Furthermore, in some necrotic cells, the disruption of the plasma membrane makes phosphatidylserine, which is found in the inner plasma membrane, accessible for staining by annexin V-FITC. Therefore, such necrotic cells also have FITC staining and PI staining (FITC+/PI+), just like the late apoptotic-like cells. These cells are, therefore, difficult to distinguish from late apoptotic-like cells using this assay. As a result of this, the FITC+/PI+ population is termed as late apoptotic-like/necrotic cells. In contrast, the FITC-/PI+ population are purely necrotic cells.

The Annexin V- FITC apoptosis detection kit (Sigma, Cat No APOAF) was used to determine whether the observed cell death due to treatment with F1 and F5 was predominantly apoptotic or necrotic. The kit manufacturer’s instructions were followed. *T. b. brucei* cells (2 × 10^5^ cells) treated at ½ × IC_50_, 1 × IC_50_ and 2 × IC_50_ for 24 hours, and untreated cells, which served as control, were washed twice with vPBS. The cells were resuspended in 250 µL of the binding buffer, then 2.5 µL of Annexin V-FITC conjugate and 5 µL of propidium iodide (PI) solutions were added to each cell suspension. The cell suspensions were incubated in the dark at room temperature for 10 minutes. For F1-treated cells, fluorescence was acquired using a Becton Dickinson (BD) LSRFortessa X-20 cell analyser with BD FACSDiva version 8.0.1 software. Both FITC and PI were excited using the blue (488 nm) laser. B 530/30 and YG 610/20 filters, and 505 LP and 600 LP mirrors, were used for FITC and PI fluorescence detection respectively. For F5-treated cells, fluorescence was acquired using a BD FACSCalibur with CellQuest Pro software due to a technical fault with the BD LSR Fortessa X-20 cell analyser. For all the experiments, least three independent experiments were carried out. Data analysis was done using FlowJo software 10.7.1. Statistical analysis (two-way ANOVA and *post hoc* comparison was done using Dunnett’s multiple comparison test with individual variances computed for each comparison) was carried out using GraphPad Prism version 9.

**Cell cycle assay**

The effect of the fractions on cell cycle progression was investigated in live cells using the cell-penetrant Hoechst 33342 nucleic acid stain as described (Mabille et al., 2021). *T. b. brucei* cells (2 × 10^5^ cells/mL) treated for 24 hours at ½ × IC_50_, 1 × IC_50_, and 2 × IC_50_ and untreated cells, which served as the control, were harvested by centrifugation at 2700 rpm (800 g) for 5 minutes and washed with PBS. The harvested cells were resuspended in PBS containing 5 µg/mL of Hoechst 33342 (Invitrogen, Cat No H1399) and incubated for 30 minutes at room temperature before acquisition using a BD FACS LSR Fortessa X-20 flow cytometer with BD FACSDiva software version 8.0.1. The UV (355 nm) laser was used to excite Hoechst and the 450/50 filter and 410 LP mirror for DAPI was used for fluorescence detection. This assay was conducted in three biological replicates. For each replicate, 10,000 events were acquired. Data analysis was carried out using the cell cycle function (Watson pragmatic model with the G1 and G2 peaks were constrained such that CV values were the same) on the FlowJo software 10.7.1. Statistical analysis (two-way ANOVA) was carried out using GraphPad Prism version 9. Dunnett’s multiple comparison test with individual variances computed for each comparison was used for post hoc analysis.

**Results**


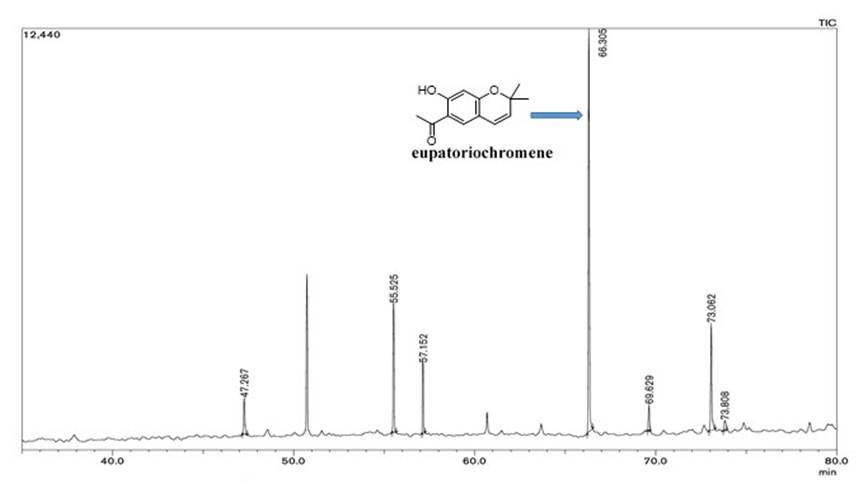


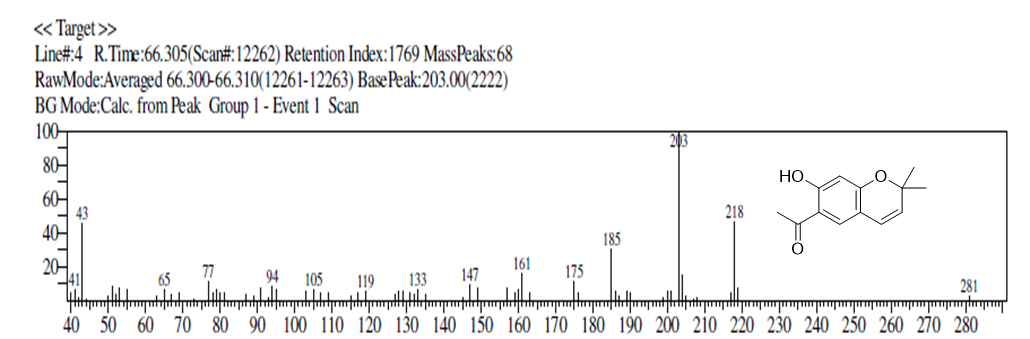


**Fig S1: Chemical characterization of F1 and F5**. Bioassay-guided fractionation of F1 gave F1/HML as the most active fraction (IC_50_ <0.0977 μg/mL). (A) The GC fingerprint of F1/HML and (B) MS of *m/z* 66.305 (eupatoriochromene). Due to the paucity of F5, no HPLC fingerprint was acquired.
